# Assessing Reassortment between Bluetongue Virus Serotypes 10 and 17 at Different Coinfection Ratios in *Culicoides sonorenesis*

**DOI:** 10.3390/v16020240

**Published:** 2024-02-02

**Authors:** Molly Carpenter, Jennifer Kopanke, Justin Lee, Case Rodgers, Kirsten Reed, Tyler J. Sherman, Barbara Graham, Mark Stenglein, Christie Mayo

**Affiliations:** 1 Department of Microbiology, Immunology, and Pathology, Colorado State University, 1601 Campus Delivery, Fort Collins, CO 80526, USA; molly.carpenter@colostate.edu (M.C.); justin.lee@colostate.com (J.L.); case1prod@gmail.com (C.R.); barb.graham@colostate.edu (B.G.); mark.stenglein@colostate.edu (M.S.); 2 Department of Comparative Medicine, Oregon Health & Science University, Portland, OR 97239, USA; kopanke@ohsu.edu; 3 Wisconsin Veterinary Diagnostic Laboratory, University of Wisconsin-Madison, Madison, WI 53706, USA; kirsten.reed@wvdl.wisc.edu; 4 Diagnostic Medicine Center, Colorado State University, Fort Collins, CO 80526, USA; tyler.sherman@colostate.edu

**Keywords:** bluetongue virus, co-infection, *Culicoides*, next-generation sequencing, reassortment

## Abstract

Bluetongue virus (BTV) is a segmented, double-stranded RNA orbivirus listed by the World Organization for Animal Health and transmitted by *Culicoides* biting midges. Segmented viruses can reassort, which facilitates rapid and important genotypic changes. Our study evaluated reassortment in *Culicoides sonorensis* midges coinfected with different ratios of BTV-10 and BTV-17. Midges were fed blood containing BTV-10, BTV-17, or a combination of both serotypes at 90:10, 75:25, 50:50, 25:75, or 10:90 ratios. Midges were collected every other day and tested for infection using pan BTV and *cox1* (housekeeping gene) qRT-PCR. A curve was fit to the ∆Ct values (pan BTV Ct—*cox1* Ct) for each experimental group. On day 10, the midges were processed for BTV plaque isolation. Genotypes of the plaques were determined by next-generation sequencing. Pairwise comparison of ∆Ct curves demonstrated no differences in viral RNA levels between coinfected treatment groups. Plaque genotyping indicated that most plaques fully aligned with one of the parental strains; however, reassortants were detected, and in the 75:25 pool, most plaques were reassortant. Reassortant prevalence may be maximized upon the occurrence of reassortant genotypes that can outcompete the parental genotypes. BTV reassortment and resulting biological consequences are important elements to understanding orbivirus emergence and evolution.

## 1. Introduction

Bluetongue virus (BTV) is the causative agent of bluetongue disease (BT) and has produced epizootics in domestic and wild ruminant populations. Due to its serious ramifications on animal health globally, BT is categorized as a “listed disease” by the World Organization for Animal Health [1,2]. Transmitted to susceptible ruminant species by the *Culicoides* biting midge (Diptera: Ceratopogonidae), BTV is the prototype virus of the genus *Orbivirus*, family *Sedoreoviridae*, in the order *Reovirales* [3,4]. Viruses of order *Reovirales* are non-enveloped and have segmented double-stranded RNA genomes that are capable of reassortment [4]. Reassortment is an important mechanism for BTV evolution and allows for its ten genomic segments to be exchanged between different parental serotypes, resulting in progeny with potentially novel phenotypes. *Culicoides* can be exposed to two or more BTV serotypes after acquiring blood meals from separate infected ruminant hosts or from a single ruminant host that is coinfected with different serotypes [5,6]. In either scenario, it is likely that the serotypes are not present in the blood at equal infectious titers. Thus, determining how BTV coinfection titers affect virus progeny outcomes is important to understanding the evolution of BTV.

With 36 BTV serotypes (24 classical serotypes and 12 genetically characterized serotypes), it is necessary to understand reassortment requirements that may limit the extent to which different parental genotypes can exchange genetic material [7,8,9]. In the field, parental genotypes must be circulating in the same geographical region at the same time (sympatric requirement) and must be able to infect the same species of host (ecological requirement). Additionally, serotypes must coinfect the same cell (coinfection requirement) and be able to package each other’s genomic segments (segment packaging requirement) within a virion. The proteins encoded by the parental viruses must be able to function together to replicate (replicative compatibility requirement). After those requirements have been met, the reassorted progeny virus must be able to compete with the parental virus (viral fitness requirement) [10]. A component of viral fitness is how well a virus can replicate and produce virus, known as virogenesis [11]. Thus, it could be conjectured that the virus strain that produces virus at a faster rate within a host would outcompete other strains in the context of available host cells to infect and host immune defenses. 

While segment 2 (2926 bp) directs conventional BTV serotype nomenclature, encoding for the viral protein 2 (VP2) that confers the serological response in susceptible hosts, the remaining nine segments are also important for BTV infection and subsequent replication [12,13]. Genomic segments 1 (3954 bp), 3 (2772 bp), and 4 (2011 bp) encode VP1 (RNA-dependent RNA polymerase), VP3 (component of the inner capsid), and VP4 (a viral capping protein), respectively [14,15,16]. Segment 5 (1769 bp) encodes NS1, which assists with viral translation and formation of microtubules, while segment 6 (1638 bp) encodes VP5, an outer shell protein that facilitates the release of the viral core into the cytoplasm [17,18,19,20]. Segment 7 (1156 bp) encodes VP7, another inner shell protein [15,16]. NS2 aids in translation and formation of viral inclusion bodies and is encoded by segment 8 (1124 bp) [17,21]. The RNA helicase, VP6, is encoded by segment 9 (1046 bp) [15]. Leaky scanning of segment 9 also yields NS4, which may have interferon antagonism properties [22,23]. Finally, segment 10 (822 bp) encodes NS3, NS3a, and NS5. NS3 and NS3a facilitate viral egress [24,25]. The newly recognized NS5 protein may have modulatory effects on the host cell response [26].

An early in vitro study investigated BTV reassortment in Vero cells coinfected at unequal multiplicities of infection (MOI) and found that the BTV serotype with the higher MOI contributed the majority of segments to the progeny virus [6]. However, there are few contemporary studies that have investigated reassortment in *Culicoides* using the more sensitive next-generation sequencing (NGS) platform. In this study, we evaluate whether varying ratios of BTV-10 and -17 fed to *Culicoides sonorensis*, the main vector of BTV in the United States, affect the genotype and reassortment outcomes of the progeny virus [3]. Additionally, we assess the rates of virogenesis to determine if the parental serotype replication rates correspond to the reassortment outcomes and to compare single infection to coinfection replication outcomes. Identifying factors that affect reassortment will inform our understanding of BTV epidemiology and emergence of novel strains. 

## 2. Materials and Methods

### 2.1. Viruses

BTV-10 California 1952 (BTV-10 ATCC) (Bluetongue virus, type 10, strain 8, ATCC^®^VR-187™) and BTV-17 Colorado 2018 (isolated by our laboratory) (BTV serotype 17 CO 2018) were used to establish BTV single and coinfections in *Culicoides sonorensis*. The strain of BTV-10 ATCC was originally isolated from a sheep in California in 1952 and passaged eight times on BHK 21 cells [27]. The strain of BTV-17 CO was isolated from the whole blood of a sheep in Colorado in 2018 on CuVaW3 cells that were derived from *Culicodes sonorensis* embryos and subsequently passaged two times on BHK 21 cells [28,29,30]. Whole genome sequences of each virus were determined previously by our lab and are available on GenBank (BTV-10 ATCC GenBank Accession MW456747-MW456756; BTV-17 CO GenBank Accession OQ798198-OQ798207) [31,32]. 

These specific strains of BTV were selected for this study because their relatively low nucleotide identities at the segment level facilitates distinguishability of genotypes by NGS (Table 1). Infectious titers for BTV-10 ATCC and BTV-17 CO were determined by the 50% tissue culture infectious dose (TCID_50_) on BHK 21 cells as described previously and calculated by the Reed–Muench equation [31,33].

### 2.2. Cells

BHK 21 cells that were acquired from ATCC (CCL-10) were used to conduct plaque isolations and propagations of BTV from homogenized infected *C. sonorensis.* BHK 21 cells were maintained in media containing Eagle’s Minimum Essential Medium (EMEM) and supplemented with 10% heat-inactivated fetal bovine serum (FBS), 10% tryptose phosphate broth, and 1% penicillin-streptomycin (10,000 U/mL). Cells were incubated at 37 °C with 5% CO_2_ and passaged at approximately 90% confluency every three to four days [31]. 

### 2.3. Culicoides Sonorensis Infection and Maintenance

AK colony *C. sonorensis* (derived from wild *C. sonorensis* in Idaho in 1973 and perpetuated by the USDA ARS, Manhattan, KS, USA) were acquired from USDA ARS [34]. Upon arrival, the *C. sonorensis* acclimatized for at least 24 h at 25 °C on a 12:12 h light cycle. Ad libitum 10% sugar water was provided [34]. At the time of infection via virus-spiked blood meal, the *C. sonorensis* were three to four days post-emergence. Mechanically defibrinated sheep’s blood (Hemostat Laboratories, Dixon, CA, USA or Lampire Biological Laboratories, Everett, PA, USA) was evaluated for the presence of BTV virus and antibodies via pan BTV quantitative real-time reverse transcription polymerase chain reaction (qRT-PCR) and cELISA (VMRD, Pullman, WA, USA) [35,36,37]. The infection experiment was divided into two studies, Study A and Study B, to accommodate limited numbers of *C. sonorensis* per experimental round. Study A included *C. sonorenesis* infection groups fed negative blood, BTV-10, BTV-17, or combined BTV-10:BTV-17 at ratios of 90:10, 75:25, or 50:50 in spiked blood meals. Study B included *C. sonorenesis* infection groups fed negative blood, BTV-10, BTV-17, or combined BTV-10:BTV-17 at ratios of 50:50, 25:75, or 10:90 in spiked blood meals. BTV was added to the blood to produce a total TCID_50_/mL of 1 × 10^5^ for each infection group within Study A and Study B (Table 2). The same BTV stocks were used in Study A and Study B. For negative control blood, the media used to suspend the virus were supplied at equal volume as the infectious blood feeding groups. Blood was provided to *C. sonorensis* via parafilm membranes on glass bell feeders that maintained blood at 37 °C using heated water circulation. Blood feeding occurred for 80 min.

After blood feeding, *C. sonorensis* were immobilized by placement into a −20° freezer for five minutes. Blood-fed *C. sonorensis* were then sorted on a modified chill table and placed in groups of approximately 100 *C. sonorensis* per container according to the BTV infection group.

For the remainder of the study, *C. sonorensis* were housed in non-treated paper tube containers (Rigid Paper Tube Corporation, Wayne, NJ, USA) with sheer panty hose stretched over the lid for air exchange and feeding. Sugar water (10% *w*/*v*) was available at all times with a cotton wick on each container. *C. sonorensis* were maintained at 25 °C ± 2 °C with a 12:12 h light cycle.

### 2.4. C. sonorensis Collections

To longitudinally evaluate virogenesis via qRT-PCR, five *C. sonorensis* were collected in triplicate from each treatment group every other day until day ten post infection or until no specimens remained. This included five to ten blood-fed midges collected immediately after blood feeding and sorting from each infection group to establish that the *C. sonorensis* were exposed to the virus at the blood feeding. At day ten post infection, according to availability, ten *C. sonorensis* from each treatment group were collected in triplicate for plaque assays. *C. sonorensis* collected were stored at −80 °C until downstream processing. Prior to nucleic acid extraction or plaque assays, *C. sonorensis* were manually homogenized with a sterile pestle in a volume of 50 µL EMEM per *C. sonorensis* (i.e., 250 µL for pools of five *C. sonorensis* and 500 µL for pools of ten *C. sonorensis*) [35]. 

### 2.5. Single and Coinfection Plaque Assays

Plaque assays were performed on the homogenized pools of ten *C. sonorensis* from each infection group. Homogenized pools were vortexed and briefly centrifuged. Further, 400 µL of the supernatant was syringe sterile-filtered (0.22 µM Millex-GV syringe filter, MilliporeSigma, Burlington, MA, USA) and further diluted in EMEM at 1:2, 1:10, 1:100, 1:1000, and 1:10,000 dilutions.

Further, 48 h prior to performing plaque assays, BHK 21 cells were seeded in 6-well plates at 1.0 × 10^5^ cells/well and maintained as described above. Immediately before inoculation with *C. sonorensis* homogenate dilutions, confluent monolayers were washed one time with 500 µL PBS (pH 7.5) per well. Each well was inoculated with 500 µL of one of the *C. sonorenesis* homogenate dilutions and incubated for 1 h at 37 °C with rocking every 15 min. The inoculum was then aspirated off the cells and the cells were washed once with PBS (pH 7.5). This was followed by an overlay with 2 mL of 3:1 BHK 21 media: 2% agarose in Earle’s Buffered Salt Solution (EBSS). The 6-well plates were incubated at 37 °C until plaques were evident by microscopy or for 96 h followed by a 1 mL overlay of 3:1 BHK media: 2% agarose in EBSS with 0.1% neutral red stain. Individual plaques were collected 8–24 h after the second overlay when plaques were visibly evident. Agarose plugs collected from discretely isolated plaques were propagated in individual wells of 48-well plates with BHK 21 cells (4.65 × 10^4^ per well). The supernatants of the propagated plaques were collected when the cytopathic effect was prominent and stored at −80 °C until further processing [35].

### 2.6. Nucleic Acid Extraction and DNase Treatment

Nucleic acid was manually extracted from all supernatants using the MagMAX Pathogen RNA/DNA kit from Applied Biosystems (Invitrogen, Carlsbad, CA, USA) according to the manufacturer’s instructions for low cell content samples. In contrast, the pools of *C. sonorensis* were processed on the KingFisher Flex magnetic particle processor (Thermo Fisher, Waltham, MA, USA) and eluted in 90 µL elution buffer, while the plaques were processed manually and eluted in 45 µL elution buffer to increase nucleic acid concentration for downstream sequencing. The *C. sonorensis* that were collected in pools of five to assess virogenesis rates via qRT-PCR were homogenized and extracted as described by Kopanke et al. [35]. The extracted homogenized *C. sonorensis* collected in pools of ten and their subsequent isolated propagated plaques were treated with TURBO DNA-*free*™ kit (Invitrogen) and incubated with 7.5 M LiCl to deplete DNA and precipitate single-stranded RNA to enrich for dsRNA as described by Kopanke et al. [35]. Afterwards, samples were treated with a 1.25 × MagMAX Pathogen RNA/DNA kit clean-up step to remove excess salts.

### 2.7. Pan BTV and cox1 qRT-PCR Assay

Extracted RNA from *C. sonorensis* homogenates was screened for BTV detection in duplicate using a universal pan BTV qRT-PCR that recognizes segment 10 as previously described [35,36,37]. 

Normalization for variations in extraction efficiency was accomplished by performing, in duplicate, a qRT-PCR based on *Culicoides* mitochondrial cytochrome c oxidase subunit 1 (*cox1*) as described by Kopanke et al. using a FAM-based probe (3′FAM-TGAATACTT/ZEN/CCTCCTTCTCTTTCTT-3IABkFQ/5′, Integrated DNA Technologies, Coralville, IA, USA) [35]. Positive and negative controls for BTV and *Culicoides cox*1 were run with each plate. Additionally, to confirm adequate DNAse treatment, a no-reverse transcriptase (no-RT) control was included. The ∆Ct method based on mean Ct values for BTV and *cox*1 for each sample (Ct_BTV_ − Ct*_cox1_ =* ∆Ct_normalized_) was applied to determine BTV Ct normalized values.

Finally, BTV-10 and BTV-17 serotype-specific qRT-PCR, as described by Maan et al., was performed on pools of *C. sonorensis* used for plaque propagation to characterize if there was presence of both BTV-10 and BTV-17 segment 2. Ct values less than 36 were considered positive for detection [38].

### 2.8. Library Preparation and Whole Genome Sequencing

Shotgun whole genome sequencing was performed on the *C. sonorensis* pools and subsequent plaques to determine BTV genotypes. Negative water controls, as well as positive controls (BTV-10 ATCC, BTV-17 CO, and BTV-10:BTV-17 at 50:50), were included with each library prep and sequencing run. Sample libraries were prepared using the NEBNext^®^ Ultra II Directional RNA Library Prep Kit for Illumina^®^ (New England BioLabs Inc., Ipswich, MA, USA), according to the Section 4 Protocols with the following modifications for BTV double-stranded RNA: (1) RNA fragmentation was performed at 90 °C for 1 min and (2) first strand cDNA synthesis was carried out for 10 min at 25 °C, 30 min at 42 °C, and then 15 min at 70 °C. NEBNext Multiplex Oligos for Illumina (96 Index Primers) were used for sample identification according to the manufacturer’s protocol. Libraries were assessed for DNA quality and concentration with Qubit high sensitivity DNA reagents on the Qubit 2.0 fluorometer (Thermo Fisher, Waltham, MA, USA) and High Sensitivity D1000 DNA screentape on the TapeStation 2200 or 4150 instruments. 

Approximately 60 samples were pooled at a time and subsequently size-selected for inserts at 300–900 base pairs with base pair size fractionation on a 1.5% agarose gel. After the appropriate region was excised from the gel, the sample was reextracted using the QIAquick Gel Extraction Kit according to the manufacturer’s protocol (Qiagen, Hilden, Germany), followed by a bead-clean up using NEBNext^®^ Ultra II Directional RNA Library Prep Kit of for Illumina^®^ Section 4.9 protocol. Pools were analyzed with the Qubit fluorometer and TapeStation instruments as described above and then quantified with the KAPA Library Quantification kit (KAPA Biosystems, Basel, Switzerland) according to the kit instructions. The NextSeq 500/550 Mid Output Kit v2.5 (300 cycles) was used to perform paired-end sequencing (2 × 150) on a NextSeq 500 sequencer instrument (Illumina, San Diego, CA, USA).

### 2.9. BTV Analysis Pipeline and Bioinformatics

Libraries were demultiplexed and then analyzed using the stenglein-lab/btv_segment_table pipeline “https://github.com/stenglein-lab/btv_segment_table (accessed on 18 May 2023)” that uses modules and infrastructure developed by the n-f core community [39,40]. This pipeline was designed to quantify the number of reads in a sample that map to each of the ten bluetongue virus genomic segments to determine the parental strain origin for each segment for assessment of reassortment. Pre-processing in this pipeline includes trimming of low-quality sequences, bases, and adapter sequences using Cutadapt with Cd-hit to collapse duplicate read pairs [41]. Reads were aligned in Bowtie 2 to the reference sequences (BTV-10 ATCC and BTV-17 CO) that we provided [42,43,44]. Default parameters were applied with the exception of the BTV reference sequences. FASTA files of BTV-10 ATCC and BTV-17 CO reference sequences used in this study were formatted and employed with the --refseq_fasta command line option. 

### 2.10. Statistical Analysis

A comparison of the rates of virogenesis between BTV-10 ATCC single infection, BTV-17 CO single infection, and BTV-10:BTV-17 50:50 in Study A (fit with an interaction of group to a 3rd degree polynomial) and Study B (fit with an interaction of group to a 2nd degree polynomial) were compared pairwise on the linear portions of the curves with calculations for standard error, z ratio statistic, and *p*-value (adjusted with Tukey’s HSD method) [45]. For ∆Ct at day zero post hoc full pairwise comparison, the conservative Scheffe adjustment to *p*-values was employed [46]. Similar comparisons were made between infection groups within a study. Statistics were performed in R version 4.2.1 with linear trends calculated with emmeans package 1.7.5 [47]. Bar graphs and heat maps of plaque genotypes were created using GraphPad Prism version 8.1.0. 

## 3. Results

### 3.1. Virogenesis of Single Infection BTV-10 ATCC and BTV-17 CO in C. sonorensis

**Figure 1:**
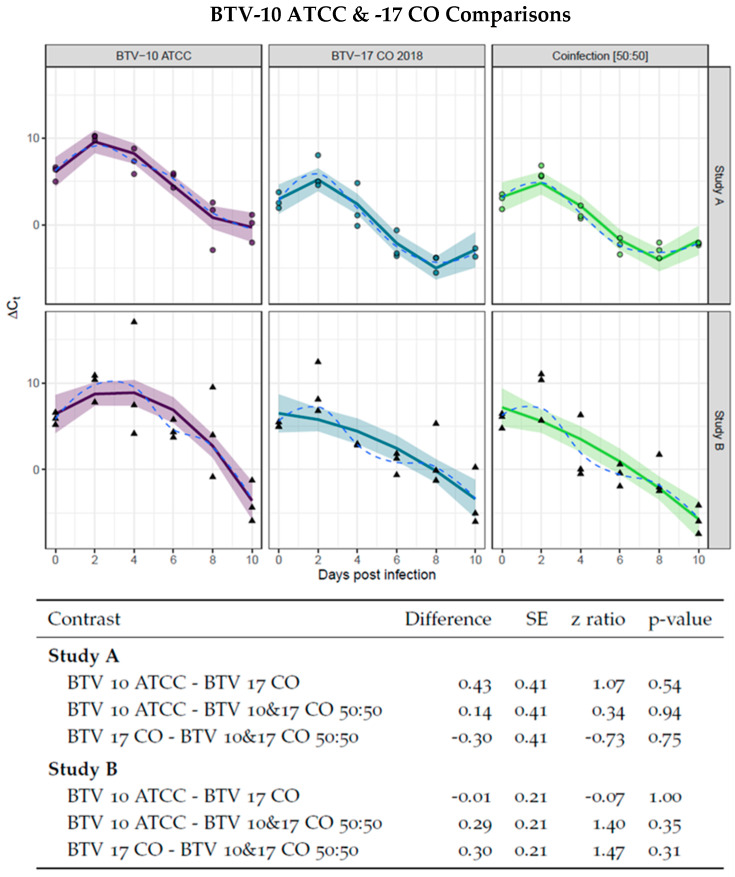
Virogenesis rates in BTV-10 ATCC, BTV-17 CO, and BTV-10:BTV-17 50:50 infection groups. BTV-10 ATCC (in purple), BTV-17 CO (in blue), and coinfection of BTV-10: BTV-17 50:50 (in green) infection groups demonstrated no statistical difference as determined by pairwise comparison of the linear portion of curve. Each point (dot for Study A and triangle for Study B) represents the ∆Ct of pool of *Culicoides* (n = 5) collected at the indicated day post infection. The ∆Ct is calculated as the difference between mean pan BTV CT and *cox1* Ct. Study A is fit with an interaction of group to a 3rd degree polynomial and Study B is fit with an interaction of group to a 2nd degree polynomial. The solid line indicates the model curve with a 95% confidence band. Standard error, z ratio statistic, and *p*-value adjusted with Tukey’s method are given for each comparison.

**Figure 2:**
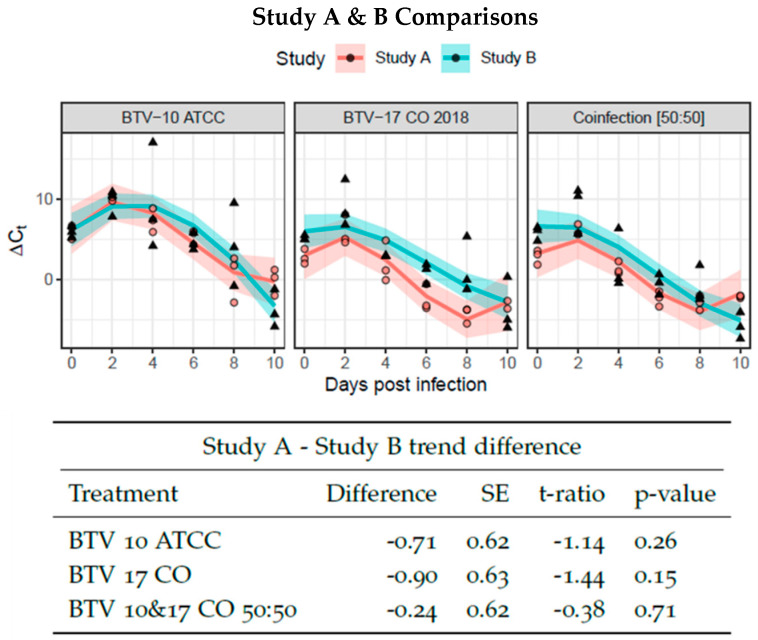
Virogenesis rates between Study A and B. BTV-10 ATCC (left), BTV-17 CO (center), and coinfection of BTV-10: BTV-17 50:50 (right) infection groups were compared between Study A and B and demonstrated no statistical difference as determined by pairwise comparison of the linear portion of curve. Each point (dot for Study A and triangle for Study B) represents the ∆Ct of pool of *Culicoides* (n = 5) collected at the indicated day post infection. The ∆Ct is calculated as the difference between mean pan BTV CT and *cox1* Ct. Study A and B are fit with an interaction of group to a 3rd degree polynomial. The solid line indicates the model curve with a 95% confidence band. Standard error, t ratio statistic, and *p*-value adjusted with Tukey’s method are given for each comparison.

First, it was necessary to establish that the colony of *C. sonorensis* can support replication of both BTV-10 ATCC and BTV-17 CO and that the serotypes have similar rates of virogenesis. This was to assess if one serotype would dominate infection as demonstrated in earlier studies [35]. To evaluate rates of virogenesis between BTV-10 ATCC and BTV-17 CO, *C. sonorensis* were provided blood meals with either BTV-10 ATCC or BTV-17 CO spiked at equal titers. The presence of BTV was assessed via Ct values of pan-BTV qRT-PCR performed on pools of five *C. sonorensis* collected in triplicated every other day and normalized by Ct values of the housekeeping gene *cox1*. The resulting ∆*Ct_normalized_* was plotted over time and fit to a curve. Pairwise comparison between the slopes of the linear portions of the curve demonstrated no significant differences, indicating that the presence of BTV RNA was similar between single infections of BTV-10 ATCC and BTV-17 CO as well as BTV-10 and -17 in 50:50 ratio infection groups. This was performed for both Study A and Study B (Figure 1). Furthermore, the single infection and 50:50 coinfection group curves were compared between Study A and Study B to ensure consistency between the studies. Pairwise comparison between Study A and Study B curves demonstrated no significant differences (Figure 2). 

### 3.2. Virogenesis of BTV-10 ATCC and BTV-17 CO at Different Coinfection Ratios in C. sonorensis

**Figure 3:**
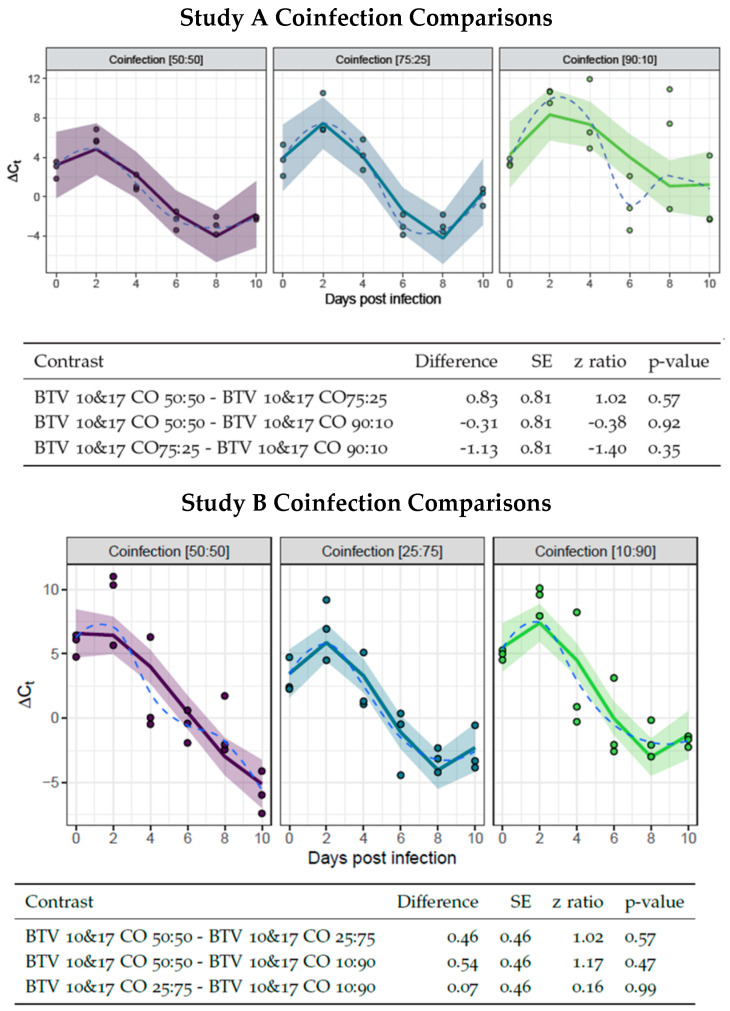
Virogenesis rates in infection groups at different ratios. Study A BTV-10: BTV-17 infection groups are represented by 50:50 (in purple), 75:25 (in blue), and 90:10 (in green). Study B BTV-10: BTV-17 infection groups are represented by 50:50 (in purple), 27:75 (in blue), and 10:90 (in green). Each point represents the ∆Ct of pool of *Culicoides* (n = 5) collected at the indicated day post infection. The ∆Ct is calculated as the difference between mean pan BTV Ct and *cox1* Ct. Study A and B are fit with an interaction of group to a 3rd degree polynomial. The solid line indicates the model curve with a 95% confidence band. The dashed line represents the mean of the data. Standard error, z ratio statistic, and *p*-value adjusted with Tukey’s method are given for each comparison.

Similar to the single infection groups, ∆*Ct_normalized_* was assessed over time for each of the different ratio infection groups. The rationale for this assessment was to evaluate whether coinfection at different ratios had synergistic or deleterious effects on virogenesis across treatment groups. If a treatment group had increased virogenesis, it would be interesting to note if that group also had a corresponding increased frequency of reassortment. However, pairwise comparisons between the linear portions of the curves of each treatment group within Study A or B determined there were no significant differences (Figure 3).

### 3.3. BTV-10 and BTV-17 Serotype Detection in Pools of C. sonorensis

To select the plaques propagated from coinfected *C. sonorensis* for sequencing, the *C. sonorensis* pools used for plaque assays were evaluated for the presence of segment 2 of both BTV-10 and BTV-17 via serotype-specific qRT-PCR. Up to ten plaques were sequenced from pools that had both serotypes represented by qRT-PCR and up to five plaques were sequenced from pools that only had one segment 2 genotype represented by qRT-PCR (if there were adequate number of plaques isolated). Notably, all pools, with the exception of pools B and C from the 25:75 coinfection group and all three pools from the 10:90 coinfection group, had both segment 2 genotypes represented by qRT-PCR (Table 3). Sequencing of additional pools was attempted; however, due to the numbers of low BTV-mapping reads, most of the pools could not be adequately characterized. 

### 3.4. Genotypes of Progeny in Plaques Propagated from Coinfected C. sonorensis

Progeny plaques isolated from the plaque assays were classified as either BTV-10 ATCC, BTV-17 CO, or a reassortant genotype as determined by sequencing results. If 90% or more of the reads were aligned to one parental serotype segment and all ten segments aligned with the same parental serotype, the plaque was classified as having the genotype of that parental serotype. If 90% or more of the reads were aligned to one parental serotype segment, but not all segments came from the same parental strain, the plaque was classified as a reassortant. Plaques that contained at least one segment that aligned to both parental serotypes (less than 90% of the reads aligned with one parental serotype) were excluded from the analysis [31,35]. Of the 111 plaques sequenced across all infection groups in this study, only nine were reassortant with the rest having identical genotypes to BTV-10 ATCC or BTV-17 CO. Coinfected pools that produced reassorted plaques were the following: Study A 75:25 pool A with 5 plaques; Study A 50:50 pools B and C with a single plaque each; Study B 50:50 pool B with a single plaque; and Study B 25:75 pool A with a single plaque. Study A coinfection group 75:25 pool A is particularly compelling as it produced five of the reassortant plaques and only contained one BTV-10 plaque (Table 3). 

### 3.5. Reassortant Segregation Ratios and Segment Combinations

**Figure 4:**
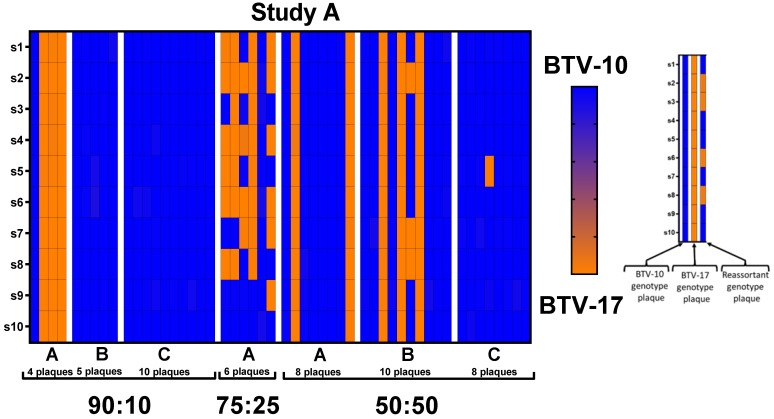

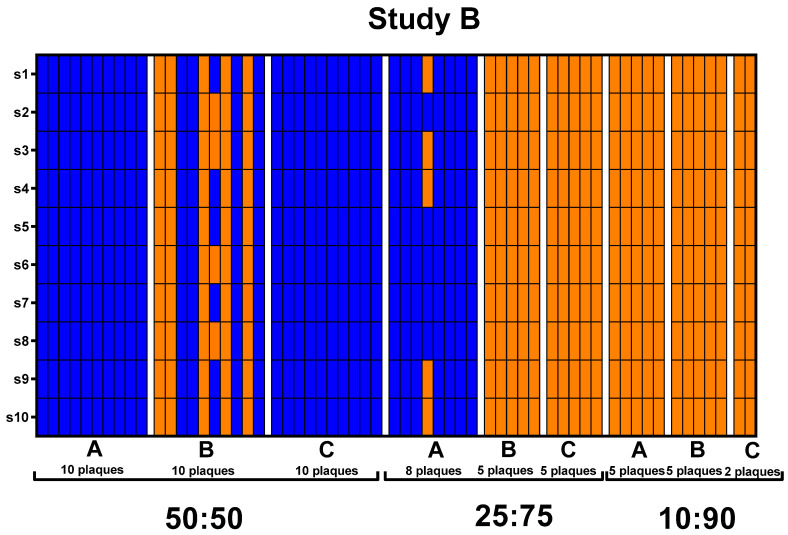
Plaque genotypes sequenced from Study A and B depicted by heat maps. Each column on the *x*-axis represents individual plaques with pools separated by white margins and indicated by letters A, B, and C. The *y*-axis represents the ten different genomic segments of BTV. Blue indicates that 100% of the sequencing reads for that segment aligns with BTV-10 ATCC. Orange indicates that 100% of the sequencing reads for that segments align with BTV-17 CO.

Results of reassortant segregation ratios, in which the first number represents the number of segments from BTV-10 ATCC and the second number represents the number of segments from BTV-17 CO (BTV-10 ATCC/BTV-17 CO), demonstrate interesting patterns in segment combinations (Table 4). Plaque genotypes from coinfection group 75:25 from Study A are particularly distinctive as the majority of plaques (five out of six sequenced plaques) are reassortants (Table 4 and Figure 4 (Study A)). It is notable that all five reassorted plaques in this pool have segments 2, 4, and 6 from BTV-17, while four and five reassorted plaques, for segments 9 and 10, respectively, align with BTV-10. Segments 9 and 10 also align with BTV-10 in most of the remainder of the reassortant plaques with a total of 7 and 8 reassortants, respectively. Additionally, all nine reassortant plaques are unique, with none displaying the same combination of genotypes (Table 4 and Figure 4). The total segregation ratio by infection group indicates the number of segments from BTV-10 ATCC and the number of segments from BTV-17 CO (BTV-10 ATCC/BTV-17 CO) from all of the reassorted plaques. Overall, contributions from BTV-10 and BTV-17 were similar with 46 total segments aligning with BTV-10 and 44 segments aligning with BTV-17 (Table 4).

### 3.6. Association between Coinfection Ratios and Plaque Genotype Outcomes

**Figure 5:**
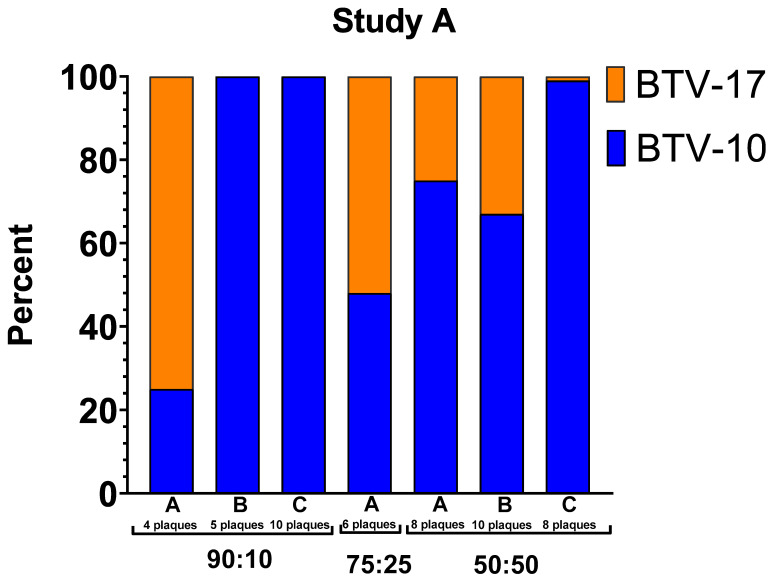

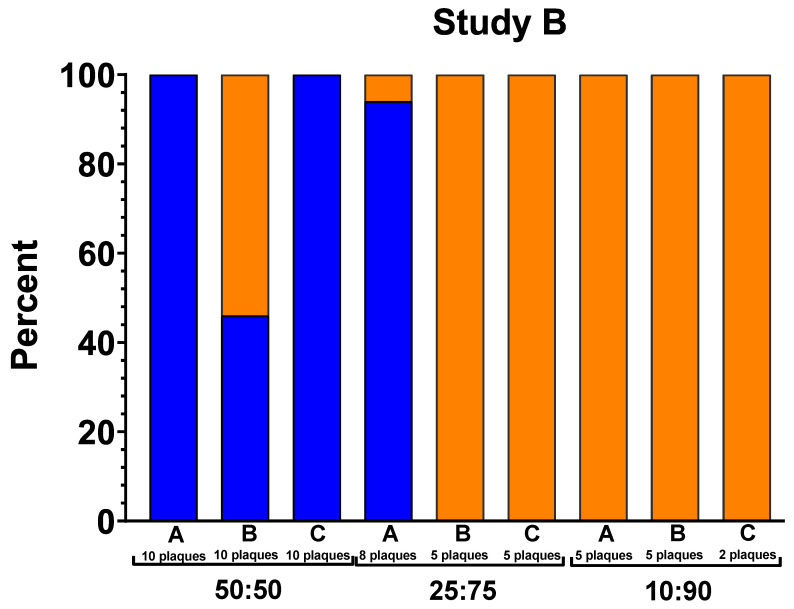
Percentage of segments that align with BTV-10 ATCC or BTV-17 CO. The *y*-axis indicates the percentage of segments that align with a parental strain out of total segments within that pool. The columns represent each pool within infection group. Blue represents BTV-10 ATCC. Orange represents BTV-17 CO.

Overall, a trend was observed in which the parental strain with the higher titer at the time of blood feeding corresponded to a higher prevalence of segments detected in the progeny virus. This is demonstrated with the plaques from the 90:10 coinfection group in Study A, mostly consisting of BTV-10 ATCC segments, and the 25:75 and 10:90 coinfection groups in Study B, mostly consisting of BTV-17 CO segments (Figure 5). However, there are notable exceptions to this trend, particularly between pools within infection groups and in the 75:25 coinfection group.

In Study A, segments from plaques propagated from pools B and C of *C. sonorensis* from the 90:10 coinfection group exclusively aligned to BTV-10, the higher titer parental serotype. However, segments from plaques in pool A of the 90:10 coinfection group aligned primarily to BTV-17 CO (75%). All plaques in this coinfection group aligned to a parental serotype with no reassortants detected (Table 4, Figure 5). In the 75:25 coinfection group, reassorted genotypes were abundant and did not correspond with the previously described trends. In this case, only 48% percent of the segments aligned with BTV-10 ATCC with 52% of the segment reads aligned with BTV-17 CO. Unfortunately, only one pool of *C. sonorensis* was available for plaque isolation in the 75:25 coinfection group due to midge survivorship. In the Study A 50:50 coinfection group, segments from both BTV-10 ATCC and BTV-17 CO were represented in each pool. Pool A had 75% BTV-10 ATCC and 25% BTV-17 CO. Pool B had 67% BTV-10 ATCC and 33% BTV-17 CO. Pool C had 99% BTV-10 ATCC and 1% BTV-17 CO (Figure 5). 

In Study B, BTV-10 ATCC segments were present in all 50:50 coinfection pools (100% in pools A and C and 46% in pool B) and in one of the 25:75 coinfection pools (94% in pool A). The remainder of the pools in the 25:75 and 10:90 coinfection groups consisted exclusively of BTV-17 segments (Figure 5). 

## 4. Discussion

Reassortment is an important mechanism for generating large genetic changes in BTV. Understanding the determinants that support or inhibit reassortment will facilitate our understanding of BTV’s evolutionary trajectory. In this study, we investigated how different coinfection ratios of parental serotypes affect progeny virus outcomes in the *Culicoides* BTV vector. 

Establishing that virogenesis rates of the two different serotypes employed in this study were similar in the colony *C. sonorensis* provided insight as to whether there was a potential for coinfection and subsequent reassortment. If the *C. sonorensis* were not competent for one of the serotypes or if the virogenesis rates were drastically different, the dominant serotype could prevent a coinfection from occurring regardless of the titer ratio. Previous studies have demonstrated the varying competencies that *Culicoides* species (and even between *C. sonorensis* colonies) have for different BTV serotypes [35,48]. Comparison of virogenesis rates between the different coinfection ratios was also performed to investigate if coinfection could exert synergistic or deleterious effects on viral replication in the *Culicoides*. Alterations in viral replication could be due to altered immune function, dissemination, or perhaps a more fit reassortant progeny. However, across all infection groups, viral replication was consistent. 

Evidence of BTV reassortment is prevalent in experimental and field studies and is predicted to have effects on transmission, pathogenicity, and the range of host diversity [49,50,51,52,53]. Reassortment has been observed in both the ruminant host and *Culicoides* vector, with the past literature indicating that *Culicoides* is a more permissive host for reassortment of BTV using electrophoretic patterns [5,49,50]. Reassortment was detected in this study; however, the occurrence of reassortment was not as robust as reported in previous in vitro or in vivo studies [5,6,31,49,50,51]. In the combined Study A and B 50:50 infection groups, only three progenies were reassortant out of the 56 plaques evaluated. In a study by Ramig et al., in vitro infection of Vero cells with BTV-10 and BTV-17 at equal MOIs resulted in 54% of the progeny analyzed being reassortant [6]. In comparison, Samal et al. demonstrated with in vivo studies using *C. sonorensis* that reassortants ranged from 7% to 78% [49]. Interestingly, Ramig et al. observed that there was quite extensive experimental variation in reassortment frequencies and ratio segregations in their in vitro infection studies and among other BTV in vivo studies. It was surmised that this was due to challenges and errors in reproducing accurate titers between studies and amplification of progeny genotypes formed early in infection [5,6,49,50]. 

The lower prevalence of reassortants in our study, which is 3/56 (5.4%) for reassorted plaques from the 50:50 pools and 9/111 (8.1%) reassorted plaques from all coinfection pools, could be related to the specific serotypes employed. Although the colony *C. sonorensis* could support replication of both BTV serotypes, there may have been limitations to the extent to which successful reassortment could occur. This could be due to the superinfection exclusion phenomenon, weak segment packaging signals, or incongruent replicative compatibility. It has been suggested that distantly related viruses may be able to overcome superinfection exclusion easier than more closely related viruses [54]. In contrast, replicative compatibility would suggest that the need for compatible virus components would favor reassortment between viruses that are more closely related [55]. Our lab performed a similar study (unpublished) in *Culicoides sonorensis* with BTV-10 ATCC and BTV-17 CA (isolated in California in 1988; GenBank Accession MT952971-MT952980) that has higher pairwise identity to investigate if genetic similarities would permit more reassortant events [28,56]. However, the caveat is that the higher the pairwise identity, the more difficult it is to distinguish genotypes with certainty. Another requirement for the successful establishment of reassortant progeny is that they must be able to compete with the parental serotype fitness. Perhaps, BTV-10 ATCC and BTV-17 CO have elevated fitness compared to most reassorted progeny outcomes, effectively making it difficult for reassortants to emerge. 

The abundance of reassortant progeny plaques in the infection group 75:25 compared to other infection groups was of interest. It could be that 75:25 was the most optimal ratio for parental fitness equivalence or perhaps the ratio simulated asynchronous infection optimal for superinfection. However, the 90:10 infection group and the 50:50 infection group both have more representation of BTV-10 ATCC in their progeny segments, which is inconsistent with those postulations. Having only had one replicate of pooled *C. sonorensis* in the 75:25 group, due to poor survivability of the midges, makes interpretations of this outcome a further challenge. Another potential explanation is that the progeny with segments 2, 4, and 6 from BTV-17 CO and segments 9 and 10 from BTV-10 ATCC have fitness gains over the parental viruses, thus they are more represented. As segments 2 and 6 both encode for outer shell proteins, perhaps there is a fitness advantage if they remain together. Infection of *C. sonorensis* with one of these reassortant progeny viruses to assess whether it elicits increased replication or a shorter extrinsic incubation period would be a fascinating follow-up study.

While not included in the analysis, ten plaques demonstrated detection of both parental genotypes in some segments. This could occur for a number of reasons. Plaque bleed over, in which two plaques may be close together and appear as one plaque but were in fact established by distinct populations, could cause a mixed genotype result. Alternatively, although BTV is considered to have selective segment packaging, this has not been thoroughly characterized and there could possibly be anomalous segment packing such as packaging of more than one copy of some segments into single particles [24]. BTV can egress from both mammalian and insect cells in lysosome-derived extracellular vesicles [57]. If extracellular vesicles contain both BTV-10 ATCC and BTV-17 CO progeny virus that infects a single cell, this could confound the plaque isolation approach for identifying reassortant progeny and also result in the observed mixed genotype. Egress and infection via extracellular vesicles could potentially confound accurate quantification of BTV via plaque assays or TCID50 and warrant further investigation. This may be reflected in the experimentation variation that Ramig et al. observed in BTV reassortment studies [6]. The potential for plaque bleed over and egress by extracellular vesicles demonstrates the limitations of using plaque isolation to distinguish progeny virus. Another challenge is plaque bias, which can occur if cells used to isolate plaques are preferentially infected by and demonstrate cytopathic effect from specific progeny genotypes. Thus, the results of this study highlight the complexities of BTV reassortment and the inherent challenges that accompany its investigation.

## Figures and Tables

**Table 1:** Nucleotide pairwise identity of BTV-10 ATCC and BTV-17 CO genomic segments.

Segment	Protein Encoded	{%} Pairwise Identity
Seg-1	VP1	96.1
Seg-2	VP2	68.8
Seg-3	VP3	97.3
Seg-4	VP4	96.3
Seg-5	NS1	96.9
Seg-6	VP5	79.5
Seg-7	VP7	95.8
Seg-8	NS2	96.2
Seg-9	VP6/NS4	96.9
Seg-10	NS3/NS3A/NS5	81.7

**Table 2:** BTV TCID_50_/mL for infection groups.

Study A Infection Group	BTV-10 TCID_50_/mL	BTV-17 TCID_50_/mL	Total TCID_50_/mL
Negative	N/A	N/A	N/A
BTV-10 ATCC	1 × 10^5^	N/A	1 × 10^5^
BTV-17 CO	N/A	1 × 10^5^	1 × 10^5^
90:10 BTV-10:BTV-17	9 × 10^4^	1 × 10^4^	1 × 10^5^
75:25 BTV-10:BTV-17	7.5 × 10^4^	2.5 × 10^4^	1 × 10^5^
50:50 BTV-10:BTV-17	5 × 10^4^	5 × 10^4^	1 × 10^5^
**Study B Infection Group**	**BTV-10 TCID_50_/mL**	**BTV-17 TCID_50_/mL**	**Total TCID_50_/mL**
Negative	N/A	N/A	N/A
BTV-10	1 × 10^5^	N/A	1 × 10^5^
BTV-17	N/A	1 × 10^5^	1 × 10^5^
50:50 BTV-10:BTV-17	5 × 10^4^	5 × 10^4^	1 × 10^5^
75:25 BTV-10:BTV-17	2.5 × 10^4^	7.5 × 10^4^	1 × 10^5^
90:10 BTV-10:BTV-17	1 × 10^4^	9 × 10^4^	1 × 10^5^

**Table 3:** *C. sonorensis* pool serotype and plaque genotype classifications.

									Genotype		
			PCR Segment 2 Genotype Detection			BTV-10 ATCC		BTV-17 CO		Reassortant	
	Infection Group	Pool	BTV-10 ATCC	BTV-17 CO	No. Plaques Sequenced	No.	%	No.	%	No.	%
Study A	90:10	A	+	+	4	1	25	3	75	0	0
	90:10	B	+	+	5	5	100	0	0	0	0
	90:10	C	+	+	10	10	100	0	0	0	0
	90:10 Total				19	16	84	3	16	0	0
	75:25	A	+	+	6	1	17	0	0	5	83
	50:50	A	+	+	8	6	75	2	25	0	0
	50:50	B	+	+	10	6	60	3	30	1	10
	50:50	C	+	+	8	7	88	0	0	1	12
	50:50 Total				26	19	73	5	19	2	8
Study B	50:50	A	+	+	10	10	100	0	0	0	0
	50:50	B	+	+	10	4	40	5	50	1	10
	50:50	C	+	+	10	10	100	0	0	0	0
	50:50 Total				30	24	80	5	17	1	3
	25:75	A	+	+	8	7	88	0	0	1	12
	25:75	B	−	+	5	0	0	5	100	0	0
	25:75	C	−	+	5	0	0	5	100	0	0
	25:75 Total				18	7	39	10	55	1	6
	10:90	A	−	+	5	0	0	5	100	0	0
	10:90	B	−	+	5	0	0	5	100	0	0
	10:90	C	−	+	2	0	0	2	100	0	0
	10:90 Total				12	0	0	12	100	0	0

**Table 4:** Segregation ratios (BTV-10 ATCC/BTV-17 CO) of genome segments from pools with reassortant genotypes.

Study	Infection Group Pool	Number of Reassorted Plaques	Seg-1	Seg-2	Seg-3	Seg-4	Seg-5	Seg-6	Seg-7	Seg-8	Seg-9	Seg-10	Total Ratio by Infection Group
A	75:25 Pool A	5	1/4	0/5	3/2	0/5	2/3	0/5	2/3	2/3	4/1	5/0	19/31
	50:50 Pool B	1	1/0	0/1	1/0	1/0	1/0	1/0	0/1	0/1	1/0	1/0	7/3
	50:50 Pool C	1	1/0	1/0	1/0	1/0	0/1	1/0	1/0	1/0	1/0	1/0	9/1
B	50:50 Pool B	1	1/0	0/1	0/1	1/0	1/0	0/1	1/0	0/1	1/0	1/0	6/4
	25:75 Pool A	1	0/1	1/0	0/1	0/1	1/0	1/0	1/0	1/0	0/1	0/1	5/5
Total	ratio by seg	9	4/5	2/7	5/4	3/6	5/4	3/6	5/4	4/5	7/2	8/1	46/44

## Data Availability

Data are contained within the article.
